# The role of mitochondrial proteases in leukemic cells and leukemic stem cells

**DOI:** 10.1002/sctm.20-0142

**Published:** 2020-08-05

**Authors:** Sara Mirali, Aaron D. Schimmer

**Affiliations:** ^1^ Princess Margaret Cancer Centre Toronto Ontario Canada; ^2^ Institute of Medical Science, University of Toronto Toronto Ontario Canada

**Keywords:** acute myelogenous leukemia (AML), cancer, hematologic malignancies, hematopoietic stem cells

## Abstract

The biological function of most mitochondrial proteases has not been well characterized. Moreover, most of the available information on the normal function of these proteases has been derived from studies in model organisms. Recently, the mitochondrial proteases caseinolytic protease P (CLPP) and neurolysin (NLN) have been identified as therapeutic targets in acute myeloid leukemia (AML). Both proteases are overexpressed in approximately 40% of AML patients. Mechanistically, CLPP and NLN maintain the integrity of the mitochondrial respiratory chain: CLPP cleaves defective respiratory chain proteins, while NLN promotes the formation of respiratory chain supercomplexes. In this review, we highlight the functional consequences of inhibiting and activating mitochondrial proteases and discuss their potential as therapeutic targets in AML.

## Graphical abstract

The mitochondrial proteases neurolysin (NLN) and caseinolytic protease P (CLPP) have been identified as therapeutic targets in acute myeloid leukemia (AML). Inhibition of NLN by R2 reduces LETM1 complex formation, respiratory chain supercomplex (RCS) assembly, and AML growth. Activation of CLPP by ONC201 or ONC212, as well as inhibition of ClpXP by A2‐32‐01, reduces oxidative metabolism and impairs AML growth.

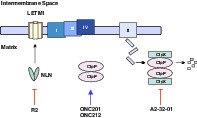

**Significance statement**

Acute myeloid leukemia (AML) is an aggressive hematological malignancy. Despite recent advances and new therapies for this disease, the prognosis for most patients with AML remains poor. Understanding the biology of this disease is important for developing new therapies. Recently, AML cells and stem cells have been shown to have unique mitochondrial properties, which can be therapeutically targeted. One of these properties is an increased reliance on the mitochondrial matrix proteases, caseinolytic protease P and neurolysin. This perspective discusses the effects of genetically and chemically dysregulating mitochondrial proteases in AML. Moreover, this study considers the potential of targeting mitochondrial proteases as a novel therapeutic strategy.

## 1 INTRODUCTION

Acute myeloid leukemia (AML) is a clinically and genetically heterogeneous hematological malignancy that is characterized by the clonal proliferation of immature blast cells.^1^ AML is maintained by a population of cells called leukemic stem cells (LSCs). Similar to normal hematopoiesis, AML is organized as a hierarchy, with LSCs at the apex.^2^, ^3^ Despite recent advances and new therapies for this disease, the prognosis for most AML patients remains poor.^4^ New therapies, such as venetoclax and azacitidine, have a remission rate of approximately 70%. However, relapse rates are high and the combination treatment is much less effective in relapsed disease.^5^, ^6^ Therefore, new therapeutic strategies, especially those that target biological vulnerabilities in LSCs are needed.

We and others have shown that targeting the unique mitochondrial vulnerabilities in AML and LSCs is a potential therapeutic strategy for this disease.^7^, ^8^, ^9^, ^10^, ^11^ Among the mitochondrial pathways that are dysregulated in AML are mitochondrial proteases and mitochondrial protein degradation. Recently, in a genetic screen to identify members of the mitochondrial proteome that are important for AML and LSC viability, we identified several mitochondrial proteases as top hits.^12^ In this perspective, we highlight two mitochondrial proteases that are important for AML and LSCs: caseinolytic protease P (CLPP) and neurolysin (NLN).

## 2 AML CELLS ARE UNIQUE IN THEIR RELIANCE ON MITOCHONDRIAL PATHWAYS

**FIGURE 1:**
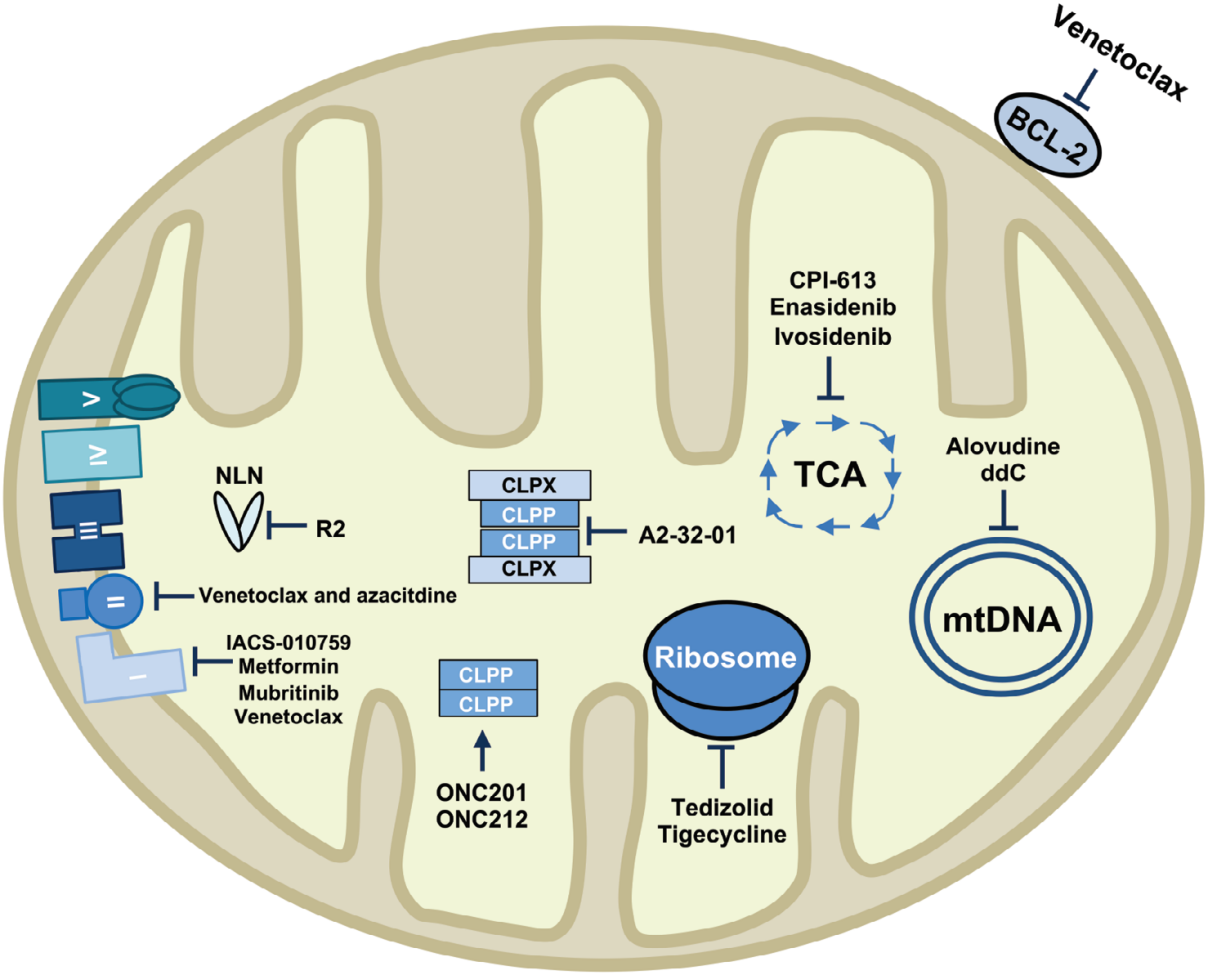
Summary of the preclinical and clinical drugs that target acute myeloid leukemia (AML) cells and leukemic stem cells (LSCs). Several drugs that target the mitochondria are currently being investigated in AML. Venetoclax binds to the antiapoptotic protein BCL‐2 and induces apoptosis.^21^ It has also been reported to inhibit respiratory chain complex I.^22^ When combined with azacitidine, venetoclax inhibits respiratory chain complex II.^10^ CPI‐613 (pyruvate dehydrogenase and α‐ketoglutarate inhibitor), enasidenib (IDH2 inhibitor), and ivosidenib (IDH1 inhibitor) inhibit enzymes within the tricarboxylic acid cycle (TCA).^20^, ^23^, ^24^ Tigecycline and tedizolid inhibit mitochondrial translation, while alovudine and ddC inhibit mtDNA replication.^8^, ^11^, ^22^, ^25^ ONC201 and ONC212 activate CLPP in the absence of CLPX.^26^ A2‐32‐01 and R2 inhibit CLPP and NLN, respectively.^12^, ^27^ IACS‐010759, metformin, and mubritinib target oxidative metabolism by inhibiting respiratory chain complex I^17^, ^18^, ^28^

AML cells and LSCs have unique mitochondrial properties compared to normal hematopoietic stem and progenitor cells (HSPCs). While normal HSPCs rely on anaerobic glycolysis, LSCs are enriched for hallmarks of oxidative metabolism and rely on oxidative phosphorylation (OXPHOS) for their survival.^7^, ^13^, ^14^, ^15^, ^16^ Moreover, compared to normal blood cells, AML cells and LSCs have higher mitochondrial mass, increased mitochondrial DNA, increased sensitivity to OXPHOS inhibitors, and decreased spare reserve capacity.^7^, ^8^, ^9^ These unique properties make targeting certain mitochondrial pathways a promising therapeutic strategy in AML: AML cells are sensitive to inhibitors of mitochondrial translation, antiapoptotic proteins, Krebs cycle enzymes, mitochondrial DNA replication, and respiratory chain complexes^8^, ^11^, ^17^, ^18^, ^19^, ^20^, ^21^ (Figure 1). Mitochondrial pathways also play a key role in AML stemness and differentiation. Increased mitophagy maintains LSCs and mitochondrial copper regulates the epigenome in leukemic cells.^29^, ^30^

The mitochondrial proteome is tightly regulated by a diverse group of enzymes called mitochondrial proteases. To date, 20 intrinsic mitochondrial proteases have been identified in the different subcompartments of the mitochondria.^31^ The functions of these proteases are not yet fully defined and some are emerging as potential therapeutic targets for malignancies. For example, the Lon protease degrades oxidized mitochondrial proteins and inhibition of LONP1 has anti‐cancer activity in colorectal cancer and melanoma, although inhibiting LONP1 does not reduce the growth of AML cells.^12^, ^32^ Likewise, inhibition of the mitochondrial protease MAP1D shows efficacy in models of colon cancer.^33^ This perspective will focus on the mitochondrial matrix proteases CLPP and NLN that have recently been reported to be overexpressed in subgroups of AML and are potentially unique therapeutic targets for this disease.

## 3 THE MITOCHONDRIAL PROTEASE CLPP AS A THERAPEUTIC TARGET FOR AML

CLPP is a mitochondrial serine protease that forms a complex with the AAA ATPase chaperone, CLPX (caseinolytic mitochondrial matrix peptidase chaperone subunit X). The ClpXP complex consists of CLPP arranged as a stacked heptameric ring with a proteolytic core capped at both ends by CLPX.^34^ CLPX's N‐terminal domain recognizes substrate proteins and feeds them into ClpXP's proteolytic core. ClpXP cleaves proteins into small peptide fragments that escape through small pores on the wall of the protease.^35^ ClpXP targets respiratory chain proteins, such as SDHA, metabolic enzymes, and proteins important for mitochondrial translation, such as ERAL1 and EFG1. Thus, ClpXP plays an important role in maintaining the respiratory chain and optimizing mitochondrial function.^12^, ^36^, ^37^, ^38^

CLPP was recently identified in a genetic screen as being important for AML cell viability. Cole et al initiated their study into CLPP by assessing its expression in primary AML samples and normal CD34+ progenitors. CLPP was overexpressed in 45% of AML samples and equally expressed in stem and bulk populations. Moreover, CLPP expression positively correlated with increased expression of genes involved in the mitochondrial unfolded protein response, suggesting that overexpression is associated with mitochondrial stress.^12^

To validate the results of the screen and to characterize CLPP's mitochondrial function, CLPP was knocked down using shRNA (short hairpin RNA). Knockdown of CLPP reduced the growth and viability of several AML cell lines with high CLPP expression, but had no effect in HL‐60 cells, which expressed low levels of CLPP. Knockdown of CLPP also targeted AML progenitors, as evidenced by reduced engraftment of TEX cells, a leukemic cell line that has stem‐like properties and hierarchical organization, into mouse marrow.^12^, ^39^ Consistent with CLPP's role in degrading SDHA, loss of CLPP increased the amount of misfolded complex II subunits and impaired complex II activity.^12^, ^38^ In line with other reports that AML cells are sensitive to reductions in OXPHOS,^7^, ^10^, ^17^ CLPP knockdown reduced oxygen consumption rates (OCR) and increased reactive oxygen species (ROS) in AML cells.^12^ The mechanism of how inhibiting complex II leads to increased ROS in AML cells remains unknown and an important future direction for investigation.

Loss of CLPP shows limited toxicity to normal tissues and CLPP deficiency in both humans and mice has not been shown to greatly affect viability. In humans, loss of CLPP causes Perrault syndrome, which is characterized by congenital hearing loss and ovarian dysfunction.^40^ Similarly, *CLPP* ^−/−^ mice have impaired hearing and are infertile. However, there was no observed effect on hematopoiesis as *CLPP* ^−/−^ mice have normal numbers and function of HSPCs and mature blood cells.^12^ Thus, loss of CLPP does not impair normal myeloid hematopoiesis, further supporting the development of CLPP inhibitors for AML. However, it should be noted that CLPX has recently been implicated in erythropoiesis and it is possible that inhibiting ClpXP may produce subtle defects in erythropoiesis.^41^ Moreover, it will be important to monitor patients treated with CLPP inhibitors for hearing loss or infertility.

β‐lactones were initially identified as potent inhibitors of bacterial CLPP through chemical proteomic screens.^42^, ^43^ The β‐lactone (3RS,4RS)‐3‐(non‐8‐en‐1‐yl)‐4‐(2‐(pyridin‐3‐ylethyl)oxetan‐2‐one (A2‐32‐01) selectively inhibited CLPP but not cytoplasmic proteases. Chemical inhibition of CLPP with A2‐32‐01 reduced the growth and viability of AML cell lines and primary patient samples. Moreover, sensitivity to A2‐32‐01 positively correlated with CLPP expression. In vivo, A2‐32‐01 impaired complex II activity and reduced engraftment of primary AML patient samples into mouse marrow in primary and secondary experiments, demonstrating a functional effect on LSCs.^12^ Although a useful tool compound, A2‐32‐01 is unstable and not suitable as a lead for a clinical candidate. Therefore, novel CLPP inhibitors based on different pharmacophores are required.

Hyperactivation of CLPP has also been shown to be an effective strategy to selectively target AML and LSCs over normal cells. In bacteria, CLPP activation causes uncontrolled degradation of proteins and death.^44^ In cancer cells, expression of a constitutively active mutant CLPP induces cell death in vitro and in vivo. Chemical activation of CLPP is also cytotoxic in cancer cells.^26^ Acyldepsipeptides (ADEPs) are a class of antibiotics that activate CLPP. ADEPs can also activate human CLPP but with limited potency.^45^, ^46^, ^47^ Recently, Ishizawa et al screened 747 clinically approved compounds to identify more potent CLPP activators and identified the imprimidones ONC201 and ONC212. ONC201 has shown promising preclinical results in solid and hematological malignancies and is currently being studied in phase I clinical trials in relapsed and refractory AML (NCT02392572) and as a maintenance therapy in AML after stem cell transplantation (NCT03932643).^47^, ^48^, ^49^, ^50^ ONC212 has shown promising preclinical results in AML.^51^ Structural analysis showed that ONC201 locks CLPP in its active, open conformation by binding at its interface with ClpX and increasing the size of CLPP's axial entrance pore. Moreover, ONC201 increased the opening of CLPP's side channels, facilitating the escape of degraded peptides.^26^, ^35^

To evaluate the anticancer effects of activating CLPP, AML cell lines were treated with ONC201 and ONC212. Both compounds reduced the growth and viability of AML cells and stem/progenitor cells but had little effect on CLPP knockout cells or cells expressing inactive mutant CLPP. Moreover, sensitivity to ONC201 positively correlated with CLPP expression in primary AML samples. Mechanistically, CLPP activated by ONC201 and ONC212 degraded respiratory chain proteins, reduced basal OCR, and increased mitochondrial ROS. Degradation of complex I subunits by ONC201 suggests that hyperactivation of CLPP changes its substrate selectivity, likely because of an increase in CLPP's axial pore size. Finally, treatment with ONC201 and ONC212 reduced tumor burden and increased survival in a xenograft model of Z138 cells, as well as in a patient‐derived xenograft model.^26^ Collectively, these findings suggest that mitochondrial proteases are tightly regulated in malignancy. Both inhibition and hyperactivation of CLPP are effective as a therapeutic approach in AML because they target OXPHOS through two distinct mechanisms. Moreover, expression of CLPP may act as a biomarker to predict treatment response as sensitivity to CLPP inhibitors and activators is associated with high levels of CLPP.^12^, ^26^

## 4 THE MITOCHONDRIAL PROTEASE NLN AS A THERAPEUTIC TARGET FOR AML

NLN is a zinc metallopeptidase with a conserved His‐Glu‐*X*‐*X*‐His sequence motif.^52^ The two histidine residues bind a zinc ion cofactor and the glutamate polarizes a water molecule, which acts as the attacking nucleophile. Its ellipsoid structure is divided by a deep cleft, forming two domains that are loosely connected at the base.^52^ The two domains move together as a hinge to close around the ligand, with domain II rotating 15° along its hinge axis and domain I rotating less than 5°.^53^

NLN acts on a diverse range of substrates that have been implicated in several biological functions, such as pain, blood pressure regulation, sepsis, and stroke.^54^, ^55^, ^56^ Its most well‐characterized function is the cleavage of the secreted tridecapeptide neurotensin. Among its many functions, neurotensin is involved in blood pressure regulation and has been implicated in sepsis.^55^ NLN cleaves neurotensin at its Pro10‐Tyr11 bond, rendering it inactive.^57^ However, NLN's physiological role and activity within the mitochondria is not well understood. Recently, the generation of a knockout mouse has shed more light on NLN's role. NLN knockout mice demonstrated heightened insulin sensitivity, increased glucose tolerance, and liver gluconeogenesis. Histological analysis of the gastrocnemius muscle revealed a significant reduction of oxidative fibers and mice exhausted earlier in endurance exercise tests. Surprisingly, despite its reported cleavage of vasoactive peptides, there was no observed effect in blood pressure regulation.^58^ These findings suggest that while NLN's role in blood pressure regulation is redundant in vivo, loss of NLN affects metabolism.

Several small‐molecule NLN inhibitors have previously been characterized. The dipeptide proline‐isoleucine was the first identified selective inhibitor of NLN. Although proline‐isoleucine is specific for NLN, it has a low affinity for the endopeptidase^59^ and must be administered at high concentrations, which is impractical for in vivo applications. Shortly after, Jiracek et al identified P33 (Pro‐Phe‐Ψ(PO_2_CH_2_)‐Leu‐Pro‐NH_2_) as a more potent and specific inhibitor of NLN.^60^ Recently, Hines et al. constructed a small allosteric inhibitor of NLN.^61^ This allosteric inhibitor (R2) binds largely though nonpolar contacts near NLN's hinge axis and locks NLN in an open, inactive conformation. Furthermore, it is predicted to be more bioavailable than P33 because it lacks phosphinic and carboxylic acid moieties.^61^

NLN was the top scoring protease in the same genetic screen that identified CLPP as a therapeutic target in AML.^12^ Using publicly available datasets, we found that NLN was overexpressed in 41% of AML samples compared to healthy controls. Moreover, NLN was equally expressed in both progenitor and bulk AML populations. Genetic knockdown of NLN reduced the growth and viability of several leukemic cell lines and targeted AML progenitors, as evidenced by colony‐formation assays and reduced engraftment of TEX cells into mouse marrow.^27^

NLN's mitochondrial function has not been well characterized to date. To gain insight into NLN's function, its mitochondrial interactors were identified using proximity‐dependent biotin labeling coupled with mass spectrometry (BioID‐MS). NLN interacted strongly with proteins involved in respiratory electron transport and respiratory chain complex assembly. Accordingly, genetic knockdown of NLN impaired oxidative metabolism and reduced the formation of large, quaternary structures called respiratory chain supercomplexes (RCS). RCS promote oxidative metabolism and are composed of respiratory chain complexes I, III, and IV.^62^, ^63^, ^64^ RCS are increased in a subset of AML patients compared to normal blood cells, and increased levels of RCS positively correlated with NLN expression.^27^

To identify how NLN is mediating the assembly of RCS, we revisited our BioID results. One of NLN's top mitochondrial interactors was the inner mitochondrial membrane protein, LETM1 (leucine zipper‐EF‐hand containing transmembrane protein 1), which has previously been implicated in RCS assembly. LETM1 forms two complexes, termed the minor and the major complex.^65^ Genetic inhibition of NLN reduced the assembly of both LETM1 complexes. Moreover, genetic knockdown of LETM1 and BCS1L, a member of the LETM1 complex and an interactor of NLN, reduced AML viability and oxidative metabolism.^27^ It is possible that LETM1 regulates mitochondrial cristae morphology, which would indirectly affect RCS assembly.^66^, ^67^ However, we found no changes in the amount of the master cristae regulator OPA1 upon NLN knockdown.^27^, ^68^ Future studies should investigate whether LETM1 can regulate cristae morphology in an OPA1 independent manner.

Chemical inhibition of NLN has also been shown to target AML cells and stem cells. Treatment of AML cells with R2 reduced viability, impaired RCS and LETM1 complex formation, and reduced OCR. In addition, R2 treatment reduced engraftment of patient derived xenograft models. R2 also targeted LSCs, as evidenced by reduced engraftment after secondary transplantation. Of note, R2 treatment did not affect the engraftment of normal cord blood cells, suggesting that chemical inhibition of NLN selectively targeted AML cells and stem cells but spared normal hematopoietic cells and progenitors.^27^

## 5 CONCLUSION

**FIGURE 2:**
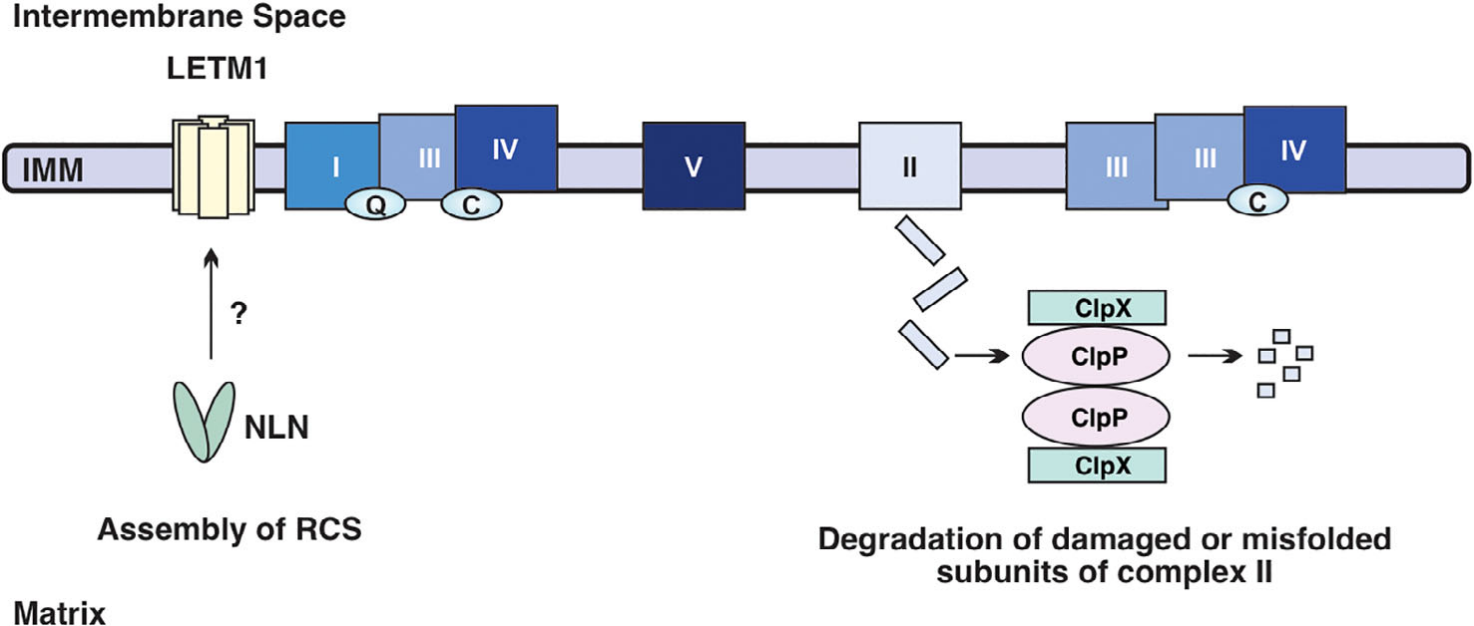
The mitochondrial respiratory chain is maintained by matrix proteases. (Left) Neurolysin (NLN) supports the formation of respiratory chain supercomplexes (RCS) through the assembly of LETM1 complexes. The mechanism by which NLN mediates LETM1 complex assembly has not been fully characterized. (Right) ClpXP degrades defective complex II subunits, preventing their aggregation and promoting complex II activity

Compared to normal hematopoietic cells, AML cells and stem cells have lower spare reserve capacity and are dependent on OXPHOS. The mitochondrial proteases, CLPP and NLN, maintain the integrity of the mitochondrial respiratory chain and promote oxidative metabolism (Figure 2). Targeting these proteases in AML has shown promising results in preclinical models but future studies will be needed to identify more stable and potent chemical inhibitors that would be suitable for clinical use. For instance, β‐lactones are unstable in human plasma, which limits their bioavailability, and attempts to stabilize these compounds reduced their ability to inhibit CLPP.^69^ Likewise, R2 is effective at high concentrations and is predicted to cross the blood‐brain barrier.^61^ In addition to developing new compounds, future work should also focus on the mechanisms underlying resistance to protease inhibitors and activators in LSCs. These findings would guide the development of more effective treatment combinations and further characterize the role of mitochondrial proteases in AML.

## CONFLICT OF INTEREST

Aaron D. Schimmer has received honorariums or consulting fees from Novartis, Jazz, Otsuka, and Takeda Pharmaceuticals and research support from Medivir AB and Takeda. Aaron D. Schimmer owns stock in Abbvie Pharmaceuticals and is named on a patent application for the use of DNT cells for the treatment of leukemia. Sara Mirali declared no potential conflicts of interest.

## AUTHOR CONTRIBUTIONS

S.M.: manuscript writing and final approval of the manuscript; A.D.S.: manuscript writing and final approval of the manuscript.
